# Use of statins in community-acquired pneumonia in intensive care settings: is there a survival advantage?

**DOI:** 10.1186/cc9687

**Published:** 2011-03-11

**Authors:** A Khanna, R Gibbs, S Webster, H Al-shather

**Affiliations:** 1 Musgrove Park Hospital, Somerset, UK

## Introduction

Use of statins in community-acquired pneumonia (CAP) and exacerbation of COPD has been widely studied [1-3]. Whilst there may be some outcome benefit with the use of statins in exacerbation of COPD, their role in CAP remains less clear. There are no studies looking at outcome benefits from statin use in patients with CAP who are admitted to the intensive therapy unit (ITU). Therefore, we conducted a retrospective cohort analysis looking at statin use and outcomes in patients with CAP admitted to our ITU.

## Methods

We retrospectively analysed 200 consecutive admissions to our ITU who had an admission diagnosis of CAP. Use of statins in those diagnosed with CAP was determined and its relation to length of stay and in-patient mortality was assessed. Baseline patient characteristics, disease severity scores, dose and type of statin prescribed were also considered.

## Results

Out of the total 200 patients with a coded diagnosis of CAP, 108 patients (54%) had CAP on notes review. Statins were prescribed in 43 (39.8%) of these patients. Statins were prescribed more often in patients >65 years old. Baseline characteristics were similar in both groups (> 60 years: 62% vs. 65%, *P *= 0.7; CURB 65 2 to 3: 48% vs. 50%, *P *= 0.8; APACHE II <10: 16% vs. 20%, *P *= 0.5; APACHE II 10 to 20: 43% vs. 42%, *P *= 1.00, APACHE II >20: 41 vs. 38, *P *= 0.7). The male:female ratio in our cohort was 1:1.3 (43% vs. 57%). Overall, in-hospital mortality in this CAP cohort was 45% (*n *= 48). This was higher than the previously reported studies [4]. We believe this represents the higher average age of the population with more accumulated co-morbidities that we cater for. Simvastatin was the most commonly prescribed statin (66% patients) in varying dosages (10 to 80 mg OD). There was no statistically significant difference in mortality between those who received statins and those who did not (55% vs. 47%, *P *= 0.29). Length of stay amongst survivors was similar in both groups (< 7 days: 58% vs. 61%, *P *= 0.7; 7 to 14 days: 39% vs. 33%, *P *= 0.4; >14 days: 3% vs. 6%, *P *= 0.4).

## Conclusions

According to this retrospective cohort study, use of statins in patients admitted to the ITU with a diagnosis of community-acquired pneumonia does not seem to provide any statistically significant survival benefit. Also, there seems to be no benefit in terms of total length of stay amongst survivors.
